# The Interfacial Dilational Rheology Properties of Betaine Solutions: Effect of Anionic Surfactant and Polymer

**DOI:** 10.3390/molecules28145436

**Published:** 2023-07-16

**Authors:** Haitao Li, Chuanzhi Cui, Xulong Cao, Fuqing Yuan, Zhicheng Xu, Lei Zhang, Lu Zhang

**Affiliations:** 1 School of petroleum engineering, China University of Petroleum (East China), Qingdao 266580, China; 2 Exploration & Development Research Institute of Shengli Oilfield Co., Ltd., SINOPEC, Dongying 257015, Chinayuanfuqing.slyt@sinopec.com (F.Y.); 3 Key Laboratory of Photochemical Conversion and Optoelectronic Materials, Technical Institute of Physics and Chemistry, Chinese Academy of Sciences, Beijing 100190, Chinaluyiqiao@mail.ipc.ac.cn (L.Z.)

**Keywords:** interface, dilational rheology, betaine, anionic surfactant, polymer, enhanced oil recovery

## Abstract

Interfacial dilational rheology is one of the important means to explore the interfacial properties of adsorption films. In this paper, the interfacial rheological properties of the mixed system of sulfobetaine ASB with a linear alkyl group and two anionic surfactants, petroleum sulfonate (PS) and alkyl polyoxyethylene carboxylate (AEC), were investigated by interfacial dilational rheology. The effect of the introduction of polymer hydrophobically modified polyacrylamide (HMPAM) on the interfacial properties of the mixed system was analyzed. In this experiment, the surfactant solution was used as the external phase and n-decane was used as the internal phase. A periodic sinusoidal disturbance of 0.1 Hz was applied to the n-decane droplets, and the changes of parameters such as droplet interfacial tension and interfacial area were monitored in real time with the help of a computer. The results show that the betaine ASB molecule responds to the dilation and compression of the interface through the change of ion head orientation, while the feedback behavior of petroleum sulfonate PS and AEC molecules embedded with oxygen vinyl groups in the molecule is diffusion and exchange between the interface and the bulk phase. Therefore, the interface film formed by ASB alone is higher, and the film formed by PS and AEC molecules alone is relatively lower. After adding two kinds of anionic surfactants to the betaine system, the ionic head of PS or AEC molecules will be attached to the positive center of the hydrophilic group of ASB molecules by electrostatic attraction and no longer adsorb and desorb with the interface deformation. The interfacial rheological properties of the compound system are still dominated by betaine, with higher dilational modulus and lower phase angle. When a small amount of HMPAM is added, or the content of hydrophobic monomer AMPS in the bulk phase is low, the intermolecular interaction at the interface is enhanced, the slow relaxation process is intensified, and the interfacial film strength is increased. As the content of AMPS further increases, hydrophobic blocks and surfactant molecules will form interfacial aggregates similar to mixed micelles at the oil-water interface, which will regulate the properties of the film by affecting the adsorption of surfactants at the interface. As long as the interfacial tension is the same, the properties of the interfacial film are the same. Based on the colloid interface science and the background of enhanced oil recovery, this study provides a reference for the field application of chemical flooding formulations.

## 1. Introduction

Interfacial rheology is one of the important means to characterize the properties of interfacial films, including interfacial shear rheology and interfacial dilational rheology. The interfacial shear rheology changes the interface shape under the premise of ensuring the inconvenience of the interface area, obtains the information of the interface film strength according to the response after applying the shear stress, and pays attention to the formation of the two-dimensional structure inside the interface film [1,2]. The interfacial dilational rheology is precisely to change the interface area under the premise of ensuring the inconvenience of the interface shape, and to feedback the interface behavior of the surface-active material according to the response of the interface tension, and to investigate the relaxation process occurring at the interface and near the interface. In contrast, the interfacial dilational rheology test avoids the interference caused by the thickness of the interface layer, and has become an important choice to describe the properties of the adsorption film and evaluate the performance of the dispersion system. In recent years, it has been widely used in food production [3,4], biomedicine [5,6], especially in the field of enhanced oil recovery [7].

Betaine is a typical zwitterionic surfactant. It has certain solubility in acidic, neutral, or alkaline aqueous solutions. It generally exists in the form of “internal salt” and has good temperature and salt resistance. Betaine surfactants have good biodegradability and low toxicity, which have now become the first choice for chemical flooding formulations in high-temperature and high-salt reservoirs [8,9,10]. A large number of research results show that betaine has unique oil-water interface properties compared with conventional surfactants. Cao et al. [11] explored the ability of betaine to reduce the oil-water interfacial tension. They found that the sulfobetaine with a straight-chain alkyl on the hydrophobic side can be mixed with the active components in crude oil to adsorb and produce a synergistic effect, reducing the oil-water interfacial tension to the order of 10^−3^ mN/m. Zhang et al. [12] investigated the strength of the oil-water interfacial film between betaine and n-decane by means of dilational rheology. It was proved that the ionic head of betaine would be continuously tiled and upright with the expansion and compression of the interface, and a large amount of energy storage would be generated inside the interfacial film. As a result, the highest value of dilational modulus can reach 80 mN/m.

In addition to betaine, ionic surfactants are usually one of the important choices for oil displacement compounds, such as petroleum sulfonates (PS). Petroleum sulfonates are cheap and widely available, which have been the backbone of chemical flooding to enhance oil recovery in ordinary medium-high permeability reservoirs in the past few decades [13,14,15]. In recent years, with the increasingly harsh development conditions, some oxyethylene (EO) groups have been embedded between the ionic head and the hydrophobic chain of the surfactant to produce anionic-nonionic surfactants. On the one hand, the introduction of the oxyethylene group makes the original ionic surfactant have certain salt resistance, making it better meet the actual needs of high salt reservoirs. On the other hand, the optimization and regulation of surface and interface properties can be achieved by adjusting the hydrophilic-lipophilic balance of surfactants and the spatial matching of hydrophilic and hydrophobic groups [16,17,18]. Similarly, the work investigating the oil-water interfacial behavior of petroleum sulfonates and anionic-nonionic surfactants by means of dilational rheology has been carried out: Chen et al. [19] characterized the demulsification efficiency of different types of demulsifiers by the change of interfacial dilational viscoelasticity of petroleum sulfonate emulsion system before and after demulsifier addition; Li et al. [20] explored the effect of the number of oxyethylene groups on the surface expansion of anionic-nonionic surfactants. They found that when the number of oxyethylene groups is large, the “flat configuration” of the polyoxyethylene chain would lead to the growth of the slow relaxation process and the increase of the dilational modulus.

It is generally believed that capillary resistance is the main factor affecting the deformation of crude oil in rock pores, and increasing capillary number (Nc) can reduce capillary resistance. Nc is mainly controlled by the flow rate and viscosity of the displacement fluid, as well as the interfacial tension between the displacement fluid and crude oil. The polymer is generally added to the oil field to achieve the bulk viscosity of the continuous phase and enhance oil recovery [21,22]. The commonly used oil displacement polymers are mainly divided into two categories: partially hydrolyzed polyacrylamide (HPAM) and hydrophobically modified polyacrylamide (HMPAM) [23]. However, it cannot be ignored that the introduction of polymer in chemical flooding formulation not only causes the change of bulk viscosity but also has an important influence on the oil-water interface behavior of surfactant molecules and the composition and properties of the interfacial film. Wang et al. [24] investigated the effect of HMPAM on the properties of ionic surfactant interfacial films by dilational rheology and interfacial tension relaxation. They found that the hydrophobic association and polymer will form a hydrophobic micro-area similar to that in an aqueous solution near the oil-water interface and form a mixed aggregate with the surfactant molecules on the interface, so the interface dilational modulus will be greatly weakened.

In general, whether it is a single surfactant system or a binary composite system formed by surfactants and polymers, the study of interfacial rheological properties has been relatively mature, but most of the surfactants in the system are single components. The compound use of surfactants is very common in the process of oil displacement [25,26,27]. Appropriate compound selection and proportion can achieve the dual effects of reducing costs and improving displacement efficiency. The use of betaine is a typical example. Sun et al. [28] used betaine as the main surfactant and petroleum sulfonate as the co-surfactant. It was found that the two were mixed and adsorbed at the oil-water interface in a certain proportion, resulting in a synergistic effect, achieving a better effect than a single component to reduce the oil-water interfacial tension. Unfortunately, so far, the characterization of the interfacial properties of the betaine compound system has only focused on the evaluation of the ability to reduce the oil-water interfacial tension, and the magnitude of the interfacial tension is only a description of the amount of adsorption on the oil-water interface. As for the strength of the adsorption film, the orientation and configuration of the surfactant molecules on the film and the interaction between each other and other information are not available, which is far from enough for the evaluation of the performance of the interfacial film.

In this paper, the interfacial rheological behavior of betaine and ionic surfactants after compounding was investigated by means of dilational rheology. At the same time, the influence of polymer on the interfacial properties of the compound system was explored, which enriched the research content of the chemical flooding formula and provided a reference for the flexible application of betaine surfactants in the oilfield.

## 2. Results and Discussion

### 2.1. Interfacial Dilational Rheological Properties of Surfactant Solution

**Figure 1:**
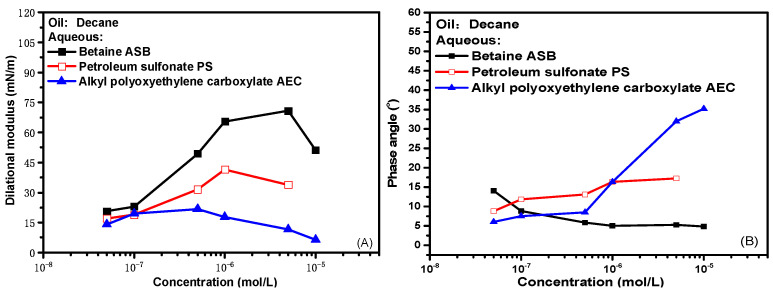
Interfacial dilational rheological properties as a function of concentration for surfactant solutions at 0.1 Hz. (**A**) Dilational modulus; (**B**) phase angle.

Figure 1 presents the experimental results of interfacial rheological properties of zwitterionic surfactant betaine ASB, anionic surfactant petroleum sulfonate PS and anionic-nonionic surfactant AEC as a function of bulk concentration at 0.1 Hz oscillation frequency, where (A) is dilational modulus and (B) is the phase angle. The dilational modulus is the direct characterization of the interfacial film strength. The dilational modulus of the three surfactants reaches a maximum with the increase of concentration and then gradually decreases. ASB has the highest extreme value, and AEC has the lowest. The difference in the maximum value is caused by the interface arrangement and configuration of the surfactant molecules. For ASB, the behavior of surfactant molecules on the interface in response to interface dilation and compression is the tile and upright of the ion head group. The main body of the diffusion exchange between the interface and the bulk phase is the solvent molecule. A large amount of storage modulus is generated in the interface film, and the film strength is high, which has been explained in detail in our previous work [12,29]. For the two ionic surfactants, the intermolecular interaction on the interface is dominated by repulsive electrostatic force. With the gradual increase of adsorption capacity, the interface deformation often leads to the continuous adsorption and desorption of surfactant molecules at the oil-water interface. The diffusion exchange behavior of surfactant molecules will greatly weaken the strength of the oil-water interface film, and the film strength is low. In contrast, the molecular size of PS is larger, and the diffusion exchange rate is slower, so the maximum dilational modulus is higher than that of AEC. Of course, after all, the dilational modulus is defined as the change of interfacial tension with the interfacial area. When the test concentration is higher than the critical micelle concentration of the surfactant, even the higher interfacial film strength will eventually be covered by the diffusion exchange behavior, and the dilational modulus of the three surfactants will eventually decrease.

The phase angle is the phase difference between the interfacial tension and the interfacial area change curve during the dilational rheology test, which is equal to the ratio of the viscous part to the elastic part of the dilational modulus [30]. The elastic part represents the interaction between surfactant molecules at the interface, and the viscous part represents the sum of a series of relaxation processes occurring at the interface and near the interface, including the interfacial rearrangement of surfactant molecules, diffusion exchange with the bulk phase, etc. With the increase of surfactant concentration, as shown in Figure 1B, the phase angle of ASB decreases slightly, and the phase angle of AEC increases significantly. It means that with the enrichment of surfactant molecules at the interface. For the betaine system, the dilational elasticity (that is, the relaxation process in the film) has always dominated the properties of the interfacial film. For the anion-non system, the diffusion and exchange of AEC molecules between the bulk phase and the interface will become more intense, which gradually dominates the properties of the interfacial film. The proportion of the viscous part begins to increase, and the phase angle increases.

### 2.2. Dynamic Interfacial Dilational Rheological Properties of Surfactant Complex System

**Figure 2:**
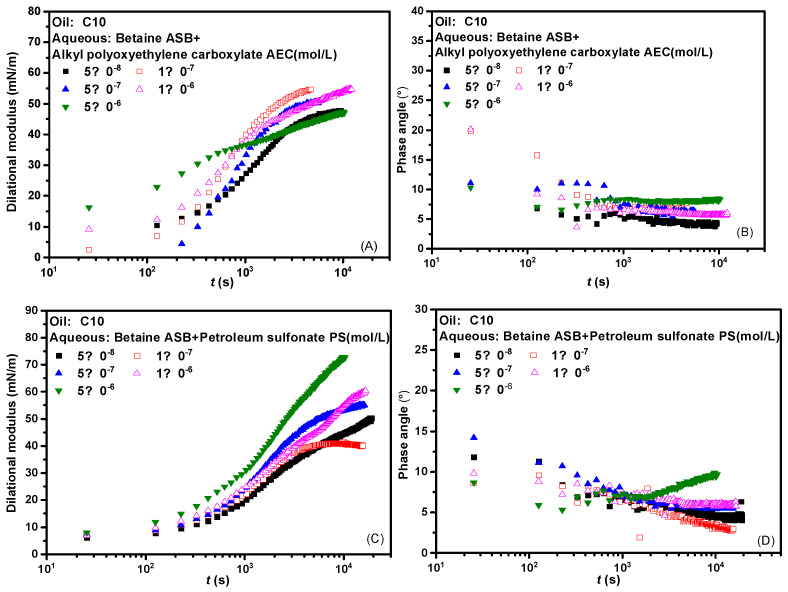
Dynamic interfacial dilational rheological properties of mixed surfactant solutions. (**A**,**C**) Dilational modulus; (**B**,**D**) phase angle.

Figure 2 presents the interfacial dilational rheological properties of the compound system changed with time where the betaine was mixed with two ionic surfactants at a molar ratio of 1:1. The dynamic modulus curve of betaine showed a trend of increasing first and then unchanged regardless of which surfactant was compounded with betaine, which means that the introduction of either anionic surfactant or anionic-nonionic surfactant did not cause changes in the adsorption state of the system. There is no dynamic process such as the change of molecular configuration or the formation and collapse of interfacial sublayer [31,32,33]. Combined with the generally low phase angle value in Figure 2B,D and the overall high interfacial dilational modulus in Figure 2A,C, we suspect that the interfacial rheological properties of the mixed system are mainly betaine after the combination of betaine and ionic surfactants. The specific verification and mechanism are carried out in subsequent experiments.

### 2.3. The Effect of Frequency on the Interfacial Dilational Rheology Properties of the Compound System

**Figure 3:**
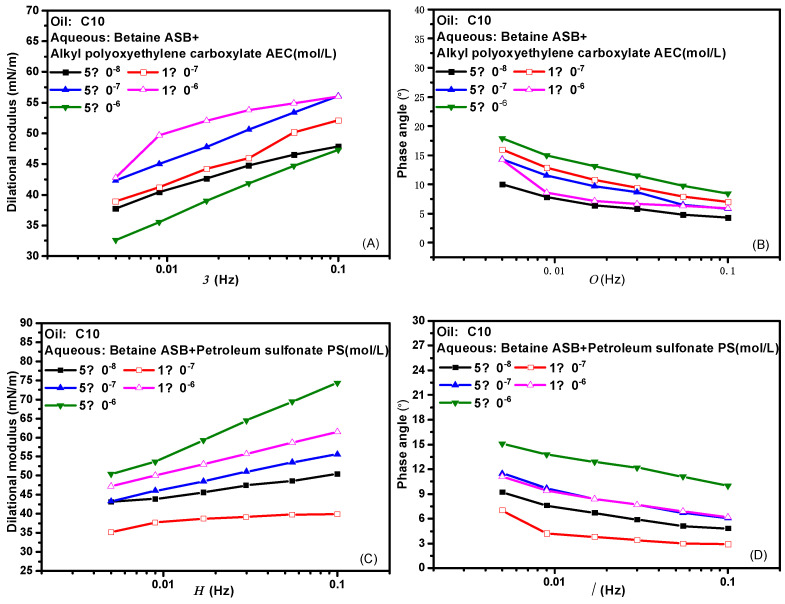
Interfacial dilational rheological properties as a function of frequency for mixed surfactant solutions. (**A**,**C**) Dilational modulus; (**B**,**D**) phase angle.

Figure 3 presents the effects of frequency on the dilational modulus and phase angle of the two composite systems. Frequency is one of the important factors affecting the interfacial dilational rheological properties. The frequency determines the difficulty of the interfacial film returning to the initial state. Similarly, different relaxation processes occurring at the interface and near the interface also correspond to their respective characteristic frequencies. In general, the weaker the frequency dependence of the surfactant, the weaker the diffusion exchange strength between the interface and the bulk phase, and the higher the interfacial film strength. Comparing the curves of the dilational modulus with frequency after betaine was compounded with two ionic surfactants, we found that the dilational modulus was almost not affected by frequency at low concentrations, and both showed a certain frequency dependence at high concentrations. The obvious slope of the curve indicates the existence of diffusion exchange, and Figure 3B,D shows that the phase angle of the two compound systems decreases slightly with the increase of frequency and is less than 20°. We believe that the interfacial compression increases the intermolecular interaction at the interface, and the contribution to the elastic modulus is equal to or slightly greater than the effect of diffusion exchange on the viscous modulus.

### 2.4. Effect of Concentration on Interfacial Dilational Rheology Properties of Complex System

**Figure 4:**
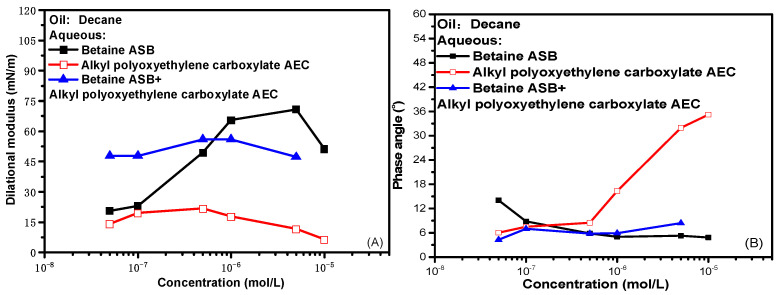

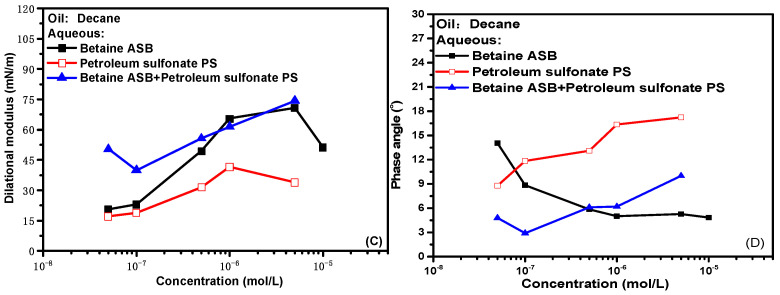
Comparisons of dilational rheological properties as a function of concentration for mixed surfactant solutions at 0.1 Hz. (**A**,**C**) Dilational modulus; (**B**,**D**) phase angle.

At 0.1 Hz frequency, the change of interfacial rheological properties of single system and compound system with concentration is shown in Figure 4. The experimental data show that the interfacial dilational parameters of the compound system are similar to those of the betaine alone, whether it is the dilational modulus or the phase angle. That is to say, as we previously conjectured, after the combination of betaine and ionic surfactants, the interfacial rheological properties of the mixed system have indeed become dominated by betaine. The hydrophilic head groups of anionic surfactants and anionic-nonionic surfactants can produce electrostatic attraction with the positive center on the betaine ion head. When the two are mixed and adsorbed at the oil-water interface, in the face of the dilation and compression of the interface, the betaine molecule still responds by adjusting the orientation of the ion head. At this time, the ionic surfactant is constrained by electrostatic attraction, the difficulty of desorption at the oil-water interface increases, and diffusion exchange no longer occurs as casually as before. In other words, the compound system at this time is actually a betaine surfactant solution with an “enlarged” ion head.

**Figure 5:**
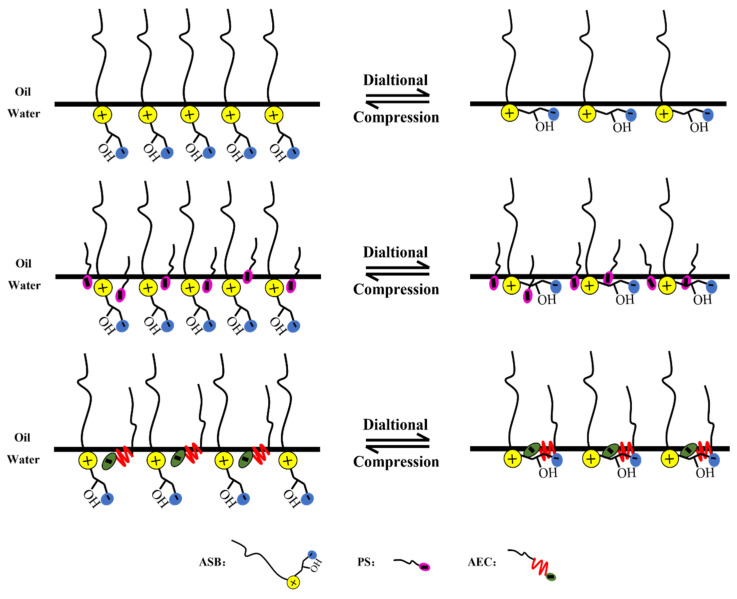
Schematic diagram of interfacial dialtional rheology mechanism of betaine single/compound system.

Comparing the maximum values of the dilational modulus in Figure 4A,C, the maximum value of the modulus becomes smaller after the betaine is compounded with the anionic-nonionic surfactant AEC after compounding with petroleum sulfonate PS, and the maximum modulus even increases slightly. This is due to the fact that the combination of surfactants is bound to bring competitive adsorption on the interface. The “flat configuration” of the oxyethylene group in AEC will occupy a certain space size [20,34,35], which reduces the number of betaine molecules adsorbed on the oil-water interface to a certain extent, and the dilational modulus will naturally decrease. Compared with AEC, PS has a smaller molecular size, and it will insert adsorption vacancies without affecting the adsorption capacity of betaine, further increasing the tightness of the adsorption film, and the dilational modulus increases. The specific mechanism is shown in Figure 5.

### 2.5. Effect of Interfacial Pressure on Interfacial Dilational Rheology Properties of Mixed System

**Figure 6:**
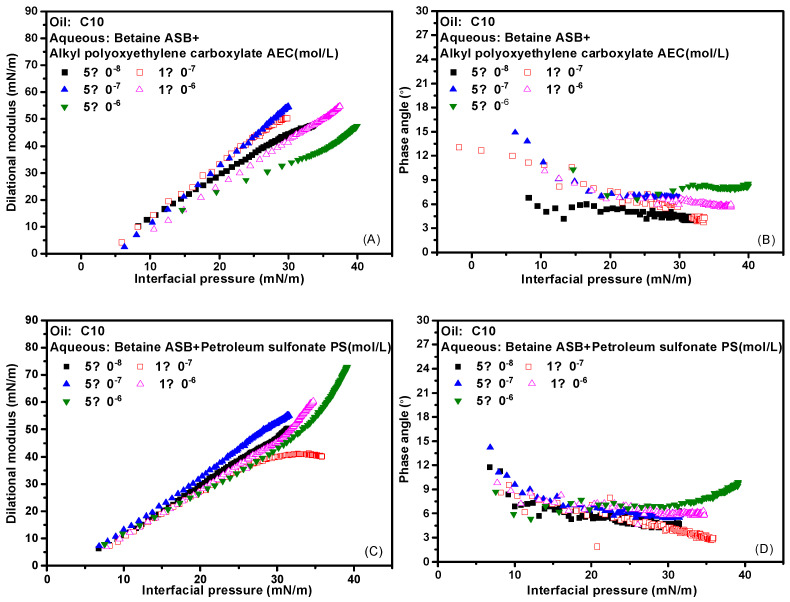
Interfacial dilational rheological properties as a function of interfacial pressure for mixed surfactant solutions at 0.1 Hz. (**A**,**C**) Dilational modulus; (**B**,**D**) phase angle.

The interfacial pressure is defined as the difference in interfacial tension before and after the adsorption of surfactant molecules. Here, the pre-adsorption refers to the oil-water interface formed by distilled water and n-decane. As mentioned above, while the interfacial tension is a direct characterization of the number of surfactant molecules adsorbed on the interface, the dilational modulus is a measure of the strength of the interfacial adsorption film. It is not only related to the adsorption amount but also determined by the intermolecular interaction and various relaxation processes. For the same surfactant solution with different concentrations, the adsorption amount and interfacial film structure are the same when the interfacial pressure is the same. If the modulus values are not much different at this time, the properties of the adsorption film are mainly determined by the relaxation process in the film; if the modulus values differ greatly, it indicates that there is a molecular escape from the interface, and the diffusion exchange process dominates the properties of the interface film. Typically, the insoluble film formed by the protein at the gas-liquid interface or the oil-water interface, the interface pressure-modulus curves at different concentrations are completely coincident [36,37,38]. Therefore, the dispersion degree of the dilational modulus-interface pressure curve of surfactant solutions with different concentrations can be used as a characterization of the contribution of the diffusion exchange process. In Figure 6A,C are the dilational modulus-interface pressure curves of the complex system formed by betaine and two ionic surfactants at different concentrations, and Figure 6B,D are the corresponding phase angle-interface pressure curves. As the interfacial pressure increases, the number of surfactant molecules adsorbed on the interface increases, while the phase angle is always very small, which means that the properties of the adsorbed film have always been determined by the relaxation process in the film. In addition, the dilational modulus-interfacial pressure curves of the two compound systems at different concentrations are relatively concentrated. Among them, the compound system of betaine and petroleum sulfonate is more compact at high concentrations, indicating that the relaxation process in the film of this system is more complicated at that time. All of these confirm our hypothesis and the inference of interface arrangement mechanism.

### 2.6. Effect of Polymer on Interfacial Dilational Rheology Properties of Compound System

**Figure 7:**
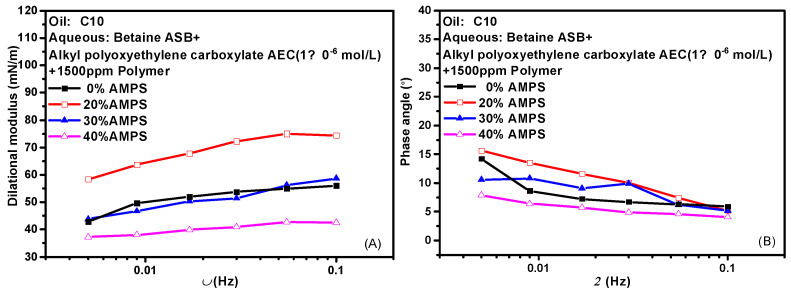

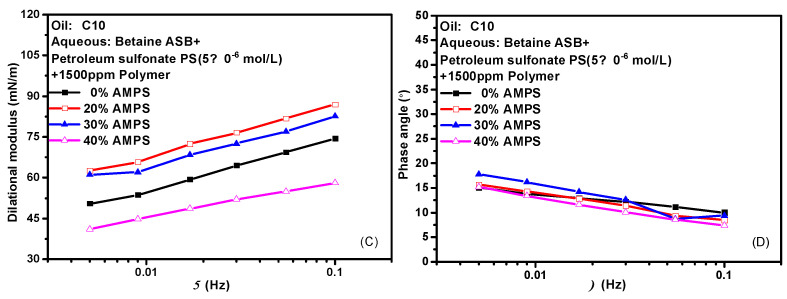
Interfacial dilational rheological properties as a function of frequency for mixed surfactant +polymer solutions at 0.1 Hz. (**A**,**C**) Dilational modulus; (**B**,**D**) phase angle.

Hydrophobically modified polyacrylamide (HMPAM) is a graft copolymer obtained by introducing a small amount of 2-acrylamido-2-methylpropanesulfonic acid (AMPS) based on hydrolyzed polyacrylamide (HPAM) [39,40,41]. The content of AMPS in the bulk phase means the degree of hydrophobic modification of the hydrolyzed polymer. HMPAM often has certain interfacial activity and can mix even compete adsorption with surfactant molecules at the oil-water interface. Figure 7 shows the variation of the interfacial dilational properties of the complex system formed by betaine and two ionic surfactants with frequency after the addition of polymers with different AMPS contents. Figure 7A,C are the dilational modulus, and Figure 7B,D are the phase angle. HMPAM is a macromolecule with a molecular weight of more than 10^7^. It has a long characteristic time of diffusion and exchange, and it is generally believed that there is only the relaxation process at the interface. However, according to the model proposed by Noskov et al. [42,43,44,45], although the diffusion and exchange process of polymer macromolecules from bulk phase to surface layer can be ignored, the diffusion and exchange process of monomer molecules between different positions of surface layer must be considered. By analyzing the modulus-frequency curves of the two composite systems, we found that in the test frequency range, as the AMPS content in the polymer increases, the value of the dilational modulus increases first and then decreases. When a small amount of hydrophobic monomers are present, the absorbed macromolecules have an almost flat two-dimensional conformation, do not form long rings and tails, and are all concentrated at the oil-water interface. The interfacial dilational compression leads to intra-film slow relaxation processes such as the deformation of polymer molecular chains. At the same time, the interaction between surfactant molecules and hydrophobic blocks at the interface is continuously enhanced, and the dilational modulus increases. With the increase of AMPS content, the surfactant molecules on the interface can form aggregates similar to mixed micelles with the hydrophobic blocks of the polymer. When the interface area increases, the surfactant molecules in the above-mixed micelles can be quickly released, and the interfacial tension gradient can be quickly eliminated in situ in the interface layer, thus greatly reducing the interfacial dilational elasticity [24,46]. It is worth noting that even if the content of AMPS reaches 40% and the dilational modulus reduced to 40 mN/m, the value of the phase angle is still very small, indicating that the strength of the interfacial film must be supplemented in some way to offset the viscous increase caused by diffusion exchange.

**Figure 8:**
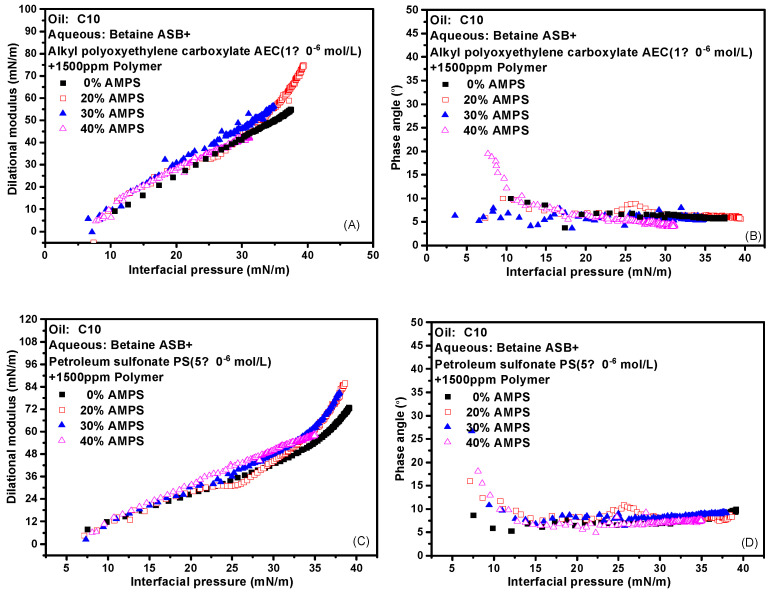
Interfacial dilational rheological properties as a function of interfacial pressure for mixed surfactant + polymer solutions at 0.1 Hz. (**A**,**C**) Dilational modulus; (**B**,**D**) phase angle.

The effect of the addition of polymer on the interface expansion parameter-interface pressure curve of the two composite systems was further investigated to verify our idea. That is, why the dilational modulus of the system began to decrease significantly after the addition of the polymer, but the phase angle was always very small, and the strength of the interfacial film was maintained or supplemented in what way? The experimental results are shown in Figure 8. The dynamic curves of different contents of AMPS almost completely overlap, and the phase angle is very small from beginning to end. A small amount of AMPS will be mixed with the surfactant molecules on the interface to increase the intermolecular interaction on the interface, which is reasonable. However, the presence of a large number of AMPS also didn’t weaken the strength of the interfacial film. We believe that the polymer regulates the properties of the film by affecting the adsorption of the surfactant at the interface. The change in the diffusion rate caused by the thickening effect has little effect on the strength of the film. That is, although different polymers are added, the properties of the interfacial film are the same as long as the interfacial tension is the same.

## 3. Experiment Section

### 3.1. Materials

**Figure 9:**
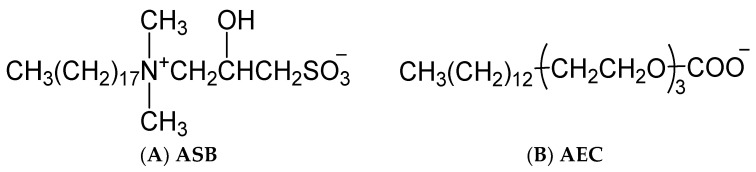
Structural formula of ASB (**A**) and AEC (**B**).

The anionic surfactant petroleum sulfonate (PS) and the zwitterionic surfactant alkyl sulfobetaine (ASB) were provided by China Petroleum Exploration and Development Research Institute. The anionic-nonionic surfactant alkyl polyoxyethylene carboxylate (AEC) was synthesized in our lab. The structural formula of ASB and AEC were shown in Figure 9. Three temperature and salt-resistant polyacrylamide polymers were supplied by Shengli Oilfield. The degree of hydrolysis of the three polymers is low, and the value is between 2 and 3. The viscosity average molecular weight is 2014, 1956, and 2052 × 10^4^ for AMPS 20%, AMPS 30%, and AMPS 40%, respectively. The decane was of 99+ mol% purity. Ultrapure water was used in the preparation of the aqueous solutions.

### 3.2. Experimental Method

The dilational rheological properties were measured on an optical dilational rheometer LSA100OEDM (Beijing Eastern-Dataphy Instrument Co., Ltd., Beijing, China). It measures the interfacial dynamic tension and dilational rheological properties by oscillating droplets and using the droplet shape analysis method. In the experiment, the periodic sinusoidal perturbations were applied to the oil droplets in the water phase, and the changes of interfacial tension and interfacial area were recorded by computer. Using a curved needle, an 8 µL oil droplet (N-decane) was squeezed into the surfactant solution and apply a sinusoidal disturbance with a frequency of 0.1 Hz and an amplitude of 1.5 µL to the oil droplet through an oscillator. At the same time, a high-speed camera is exploited to capture the profile of the oil drop in the surfactant solution and transmit it to the data acquisition computer in real time. The profile is fitted by the Laplace equation; the dynamic value of the IFT with the interface area changed will be obtained. Until the IFT value no longer changed within 30 min, the test is considered complete. Details of the instrumentation and experimental procedure have been published previously. All experiments are conducted at 25 ± 0.5 °C.

### 3.3. Theoretical Background

When the interface is periodically compressed and expanded, the interfacial tension also changes periodically. The dilational modulus *ε* is defined as the ratio of the change of interfacial tension *γ* to the change of relative interfacial area *A* [24,33,47,48]. That is:

(1) $$\varepsilon =\frac{d\gamma }{dlnA}$$

For viscoelastic interface, there is a certain phase difference *θ* between the periodic change of interfacial tension and interfacial area, which is called the phase angle of dilational modulus. The dilational elasticity $\varepsilon_{d}$ and the dilational viscosity $\varepsilon_{\eta }=\left(\omega \eta_{d}\right)$ can be calculated by the absolute value and phase angle of the dilational modulus [24,33,49,50]:

(2) $$\varepsilon_{d}=\left|\varepsilon \right|cos\theta$$

(3) $$\eta_{d}=\frac{\left|\varepsilon \right|}{\omega }sin\theta$$

## 4. Conclusions

In this paper, the interfacial dilational rheology properties of zwitterionic surfactant betaine ASB were investigated by means of dilational rheology with small amplitude low frequency oscillation. The effects of anionic surfactants and polymer on interfacial properties were investigated. The experimental results show that:

(1) The oil-water interfacial film strength of zwitterionic surfactant betaine is higher. The interfacial dilational modulus can reach 75 mN/m, which is much larger than that of ionic surfactants, while the phase angle is always lower than 15°, and smaller than conventional surfactants. The ionic head size of betaine is large, and the surfactant molecules adsorbed on the film will change their orientation with the variation of interface area, rather than escaping to the water phase. Most of the external work is stored in the interface film.

(2) The addition of anionic surfactant has little effect on the interfacial rheology properties of betaine. For the mixed system composed of ASB and PS or AEC, the interfacial dilational rheological properties are still dominated by ASB, and the modulus-interfacial pressure curves are relatively concentrated. The maximum dilational modulus can reach about 70 mN/m, phase angle is less than 18°. The difference is that the molecular size of petroleum sulfonate PS is small, which can be inserted into the adsorption vacancy of ASB molecules. The adsorption capacity on the interface is further increased, the intermolecular interaction is enhanced, and the film strength is increased. The molecular size of anionic-nonionic surfactants with oxygen-containing vinyl groups in the molecule is relatively large, which will compete with ASB molecules for adsorption. The number of surfactant molecules on the interface decreases, and the film strength decreases slightly.

(3) The addition of polymer has little effect on the interfacial rheology properties of betaine compound system. The content of different AMPS monomers does not affect the change trend of the interfacial tension of the system, although with the increase of concentration, mixed aggregates will be formed at the interface. The difference is that the presence of a small amount of AMPS can be mixed with the surfactant molecules at the oil-water interface. The stacking and winding of the polymer segments at the interface will enhance the intermolecular interaction, resulting in a slow relaxation process in the film, and the interfacial film strength increases. The enrichment of a large amount of AMPS at the interface will regulate the properties of the film by affecting the adsorption of surfactants at the interface. Even if the polymer with different AMPS content is added, the properties of the interfacial film are the same as long as the interfacial tension is homology.

## Data Availability

Due to privacy temporarily do not share data.
